# Low incidence of recurrent Buruli ulcers in treated Australian patients living in an endemic region

**DOI:** 10.1371/journal.pntd.0006724

**Published:** 2018-08-13

**Authors:** James W. Wynne, Timothy P. Stinear, Eugene Athan, Wojtek P. Michalski, Daniel P. O’Brien

**Affiliations:** 1 CSIRO, Australian Animal Health Laboratory, Geelong, Victoria, Australia; 2 Department of Microbiology and Immunology, University of Melbourne, Melbourne, Victoria, Australia; 3 Doherty Applied Microbial Genomics, Peter Doherty Institute for Infection and Immunity, University of Melbourne, Melbourne, Victoria; 4 Department of Infectious Diseases, Barwon Health, Geelong, Victoria, Australia; 5 School of Medicine, Deakin University, Geelong, Victoria, Australia; 6 Department of Medicine, Royal Melbourne Hospital, University of Melbourne, Melbourne, Victoria, Australia; Swiss Tropical and Public Health Institute, SWITZERLAND

## Abstract

We examined recurrent Buruli ulcer cases following treatment and assumed cure in a large cohort of Australian patients living in an endemic area. We report that while the recurrence rate was low (2.81 cases/year/1000 population), it remained similar to the estimated risk of primary infection within the general population of the endemic area (0.85–4.04 cases/year/1,000 population). The majority of recurrent lesions occurred in different regions of the body and were separated by a median time interval of 44 months. Clinical, treatment and epidemiological factors combined with whole genome sequencing of primary and recurrent isolates suggests that in most recurrent cases a re-infection was more likely as opposed to a relapse of the initial infection. Additionally, all cases occurring more than 12 months after commencement of treatment were likely re-infections. Our study provides important prognostic information for patients and their health care providers concerning the nature and risks associated with recurrent cases of Buruli ulcer in Australia.

## Author summary

*Mycobacterium ulcerans* (*M*. *ulcerans*) causes a necrotising infection of skin and soft-tissue known as Buruli ulcer. Since the regular use of antibiotics for Buruli ulcer treatment in Australian populations was introduced at the turn of the century, treatment success rates have been very high. However there is no information from the Australian setting on the risk of recurrent disease following treatment and assumed cure, despite this being important prognostic information for patients, their families and health-care providers. Furthermore, it is also not known if recurrent disease represents late relapse of the initial treated infection or a subsequent new infection. In our study we have shown for the first time in Australian patients living in an endemic area that the incidence of recurrent Buruli ulcer following treatment and healing is low, and that this risk is similar to the estimated risk of primary infection within the general population of the endemic area. Furthermore, we have used clinical, treatment and epidemiological data supported by genomic information of *M*. *ulcerans* organisms to determine that the majority of recurrent lesions appear to result from re-infection. This suggests that for a proportion of treated patients’ acquired protective immunity against the development of recurrent *M*. *ulcerans* disease does not develop from their initial infection.

## Introduction

*Mycobacterium ulcerans* (*M*. *ulcerans*) causes a necrotising infection of skin and soft-tissue known as Buruli ulcer.[1] Since the regular use of antibiotics for Buruli ulcer treatment in Australian populations was introduced at the turn of the century, treatment success rates have been very high.[2–4] Disease cure has assumed to occur if lesions have healed and there have been no recurrent lesions within 12 months of commencing treatment.[1,5] However, disease recurrence is known to occur.[6] At present there is no information from the Australian setting on the risk of recurrent disease following treatment and assumed cure, despite this being important prognostic information for patients, their families and health-care providers. Furthermore, it is also not known if recurrent disease represents a late relapse of the initial treated infection or a subsequent re-infection. Clarifying this issue may shed some light on the effectiveness of current treatments if recurrent lesions represent late disease relapse. On the other hand, if they represent re-infection, this may shed some light on the effectiveness of an individual’s immunity against new infections following eradication of an initial *M*. *ulcerans* infection, as well as ongoing transmission risk in the community. For the first time, whole genome sequencing has recently been used to examine this issue in four cases of recurrent *M*. *ulcerans* disease in Benin, Africa, and suggested that three of the cases represented disease relapse and one re-infection.[6]

The aim of our study was to determine the risk of recurrent *M*. *ulcerans* lesions following treatment and assumed cure in an Australian population and to use whole genome sequencing techniques combined with clinical, treatment and epidemiological data to determine whether recurrent lesions represented late disease relapse or re-infection.

## Methods

All confirmed *M*. *ulcerans* cases managed at Barwon Health, a tertiary referral institution in Victoria, Australia, from 1/1/1998-31/12/2016 were included in the study. A *M*. *ulcerans* case was defined as the presence of a lesion clinically suggestive of *M*. *ulcerans* plus any of (1) a culture of *M*. *ulcerans* from the lesion, (2) a positive PCR from a swab or biopsy of the lesion, or (3) histopathology of an excised lesion showing a necrotic granulomatous ulcer with the presence of acid-fast bacilli (AFB) consistent with acute *M*. *ulcerans* infection.

Recurrence was defined as a new *M*. *ulcerans* lesion appearing after the original lesion had healed that was culture positive for *M*. *ulcerans* and occurred ≥ 12 months after initial treatment. Patients were not actively followed up after 12 months from treatment commencement, therefore diagnosis of recurrence relied upon self-presentation or referral to our health service.

A ‘significant risk’ of relapse following initial treatment was defined as a) those who had surgery without at least 2 weeks of known effective combination antibiotics based on our published risk of relapse of 32% in those who have had surgery alone,[7] and our published treatment success rates in those who have surgery combined with at least 14 days of antibiotics)[8], or b) those who had antibiotics alone but did not complete the recommended 56 days duration of known effective combination antibiotics.[9]

Where available, whole genome sequencing and single nucleotide polymorphism (SNP) analysis was performed to examine genetic relationships between pairs of isolates from the same patient (two patients did not have paired isolates available). A total of 10 isolates, derived from five patients with recurrent disease, were subjected to whole genome sequencing (Table 1). Whole genome sequencing was performed as previously described.[10] Reads were then mapped against the *M*. *ulcerans* Agy99 genome [11], including the pMUM001 plasmid [12] and core SNPs across the 10 isolates identified using Samtools. Whole genome SNP analysis was also performed on an additional six previously sequenced *M*. *ulcerans* isolates obtained from the same endemic region (Bellarine Peninsula) [13].

**Table 1:** Patient characteristics associated with paired isolates from initial and recurrent episodes of *M*. *ulcerans* disease in Barwon Health Cohort 1998–2016.

Pair number	Isolate	Date of diagnosis	Time between diagnosis of lesions (months)	Age at diagnosis (years)	Gender	Site of lesion	Type of lesion	WHO category	Treatment	Significant risk of relapse following treatment of initial lesion	Proposed re-infection or relapse
1	mu614	5/12/11	44	55	M	Left leg	Ulcer x 2	3	R + Cp 37D	Yes	Re-infection
	mu_UK35	17/8/15		58		Left leg	Oedema	2	R + Cla 84D. Surgical debridement D91 atbs (cultures overgrown)		
2	mu327	06/12/2011	12	24	M	Right leg	Ulcer	1	Rif + Cp 56D	No	Relapse
	mu432	27/11/2012		25		Right leg	Ulcer	1	Nil		
3	mu77	29/9/2004	46	87	F	Right forearm	ulcer	1	R + Cp for 90D. Excision and primary closure D10 atbs (Positive margins, culture not done).	No	Re-infection with same genotype
	mu489	17/7/2008		91		Left ankle	ulcer x 2	3	Cp + Cla for 85D. Excision + SSG D4 atbs. (Culture positive)		
4	mu146	22/6/06	72	44	M	Left arm	ulcer	1	Cla for D40. Excision + closure D9 atbs. (margins negative, culture not done)	Yes	Re-infection
	mu403	6/7/12		50		Left elbow	ulcer	1	R + Cla for 56D.		
5	mu382	21/5/12	16	75	M	Left wrist + forearm	Oedematous	3	R + Cp for 100D. Debridement D25 atbs (Margins positive but culture negative).	No	Re-infection
	mu487	30/9/13		77		R lower leg	ulcer	1	R + Cla for 56D. Debridement D56 (margins Positive, culture negative)		
6	Not available	10/10/05	41	86	F	Right buttock	Ulcer	1	Excision + closure. Surgical margins positive.	Yes	Re-infection
	Not available	3/4/09		89		Left leg	Ulcer	1	Excision + closure. R + Cp for 28D.		
7	Not available	20/10/05	68	36	M	Right leg	Ulcer	1	R + Cla for 14D. Excision + closure D4 atbs (Margins negative, culture not done).	No	Re-infection
	Not available	4/7/11		42		Left knee	Ulcer	1	R + Cp for 56D.		

M: male, F: female, R: rifampicin, Cp: ciprofloxacin, Cla: clarithromycin, D: days, atbs: antibiotics, SSG: split skin graft

Data was collected prospectively using Epi-info 6 (CDC, Atlanta, GA, USA) and analysed using STATA 12 (StataCorp, College Staton, TX, USA).

### Ethics

This study was approved by the Barwon Health Human Research and Ethics Committee. All previously gathered human medical data were analysed in a de-identified fashion.

## Results

A total of 426 patients with *M*. *ulcerans* were managed at Barwon Health during the study period and included in the analysis. The median age was 57 years (IQR 37–73 years) and 225 (52.8%) were male. Thirty-four (8.0%) patients had diabetes and 35 (8.2%) were immune suppressed. Lesions were classified as World Health Organization (WHO) category one for 79.3%, category two for 10.6% and category three for 10.1% of lesions. The clinical type of lesion was classified as an ulcer for 85.1%, nodule for 6.1%, oedematous for 7.8% and plaque for 0.9%. The median duration of symptoms prior to diagnosis was 42 days (IQR 28–75 days).

**Fig 1:**
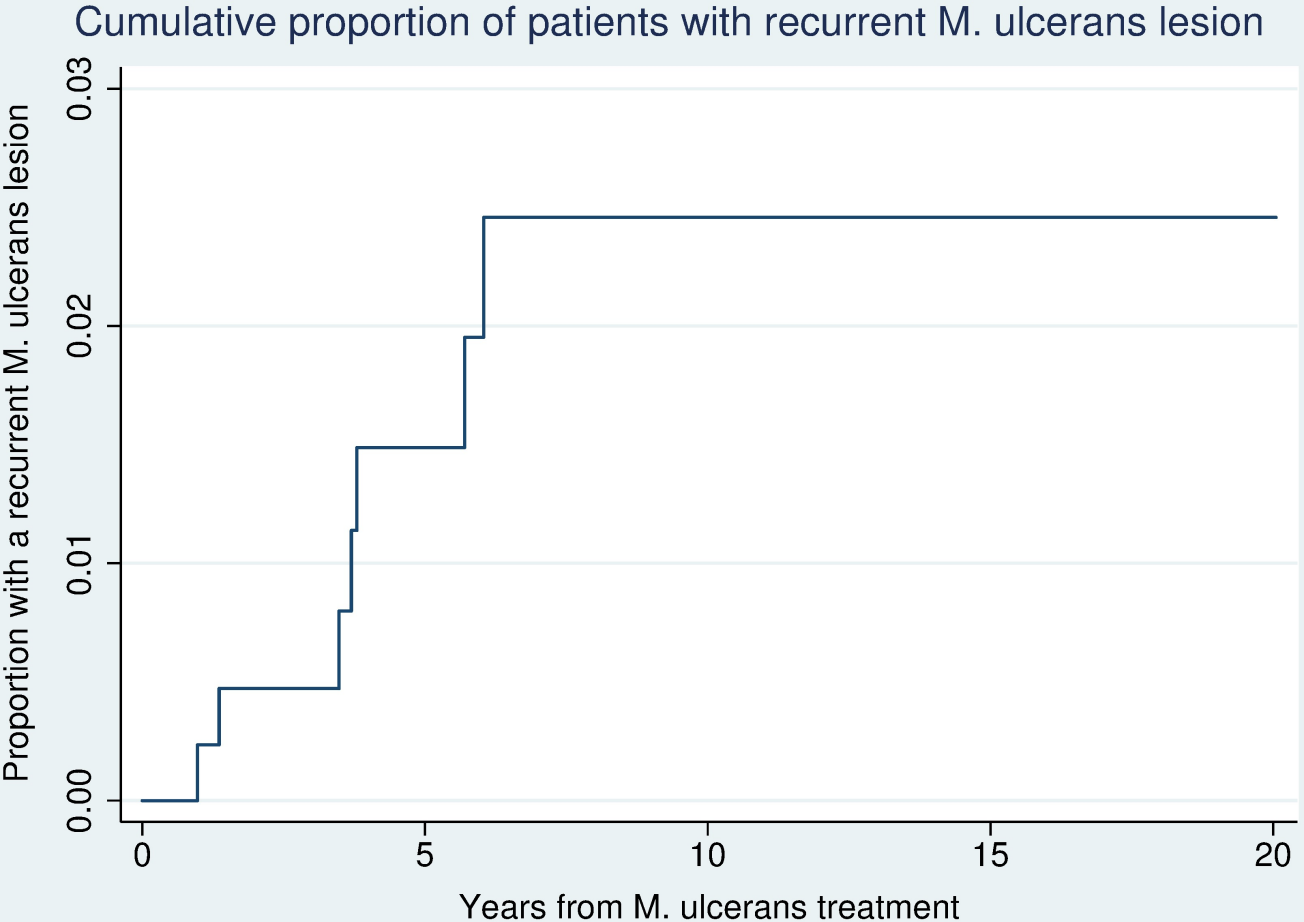
Cumulative proportion of patients with recurrent *M*. *ulcerans* lesions in the Barwon Health cohort 1998–2016.

Of this cohort, seven (1.6%) patients were diagnosed with a recurrent lesion (Table 1). This was over a combined follow-up time since commencement of treatment until the time of study analysis (12/4/18) of 2813 years, with a median follow-up time of 5.7 years (IQR 3.3–9.4 years). The rate of a recurrent lesion was 2.81 per 1000 person years (95% CI 1.19–5.22 per 1000 person years) (Fig 1). There were no significant differences in the baseline characteristics between those with a recurrence and those without a recurrence. (Table 2)

**Table 2:** Comparison of patient characteristics at diagnosis of initial *M*. *ulcerans* lesion stratified by the occurrence of a recurrent lesion.

Variable	Recurrence	No Recurrence	p-value
Gender			
Male	5	220	0.32
Female	2	199	
Median Age (years;IQR)	55 (36–86)	57 (37–73)	0.61
Diabetes	1	33	0.49
Immune suppressed	1	34	0.56
WHO category			
One	5	318	0.20
Two	0	43	
Three	2	39	
Lesion Type			
Ulcer	6	355	0.83
Nodule	0	26	
Oedema	1	32	
Plaque	0	4	
Median duration of symptoms prior to diagnosis (days;IQR)	36 (21–56)	42 (28–75)	0.43

The recurrent lesions occurred a median 44 months (IQR 16–68 months) after treatment commenced for the initial lesion; 5/7 recurrences occurred at least 3.4 years from the initial lesion. Four (57%) recurrences were on a completely separate limb and side of the body, one was on the same limb but different region of that limb and 2 were on the same limb and in the same region.

Treatment of the initial lesion involved surgery alone for 1 patient, antibiotics alone for 2 patients, and antibiotics combined with surgery for 4 patients (Table 1). According to the initial treatment, 3/7 (43%) patients were assessed as having a ‘significant risk’ of relapse; patient #1 had only 37 days of combined antibiotics alone, patient #4 had excision combined with antibiotic monotherapy with clarithromycin, and patient #6 had excision alone without adjunctive antibiotics and had positive surgical margins.

**Fig 2:**
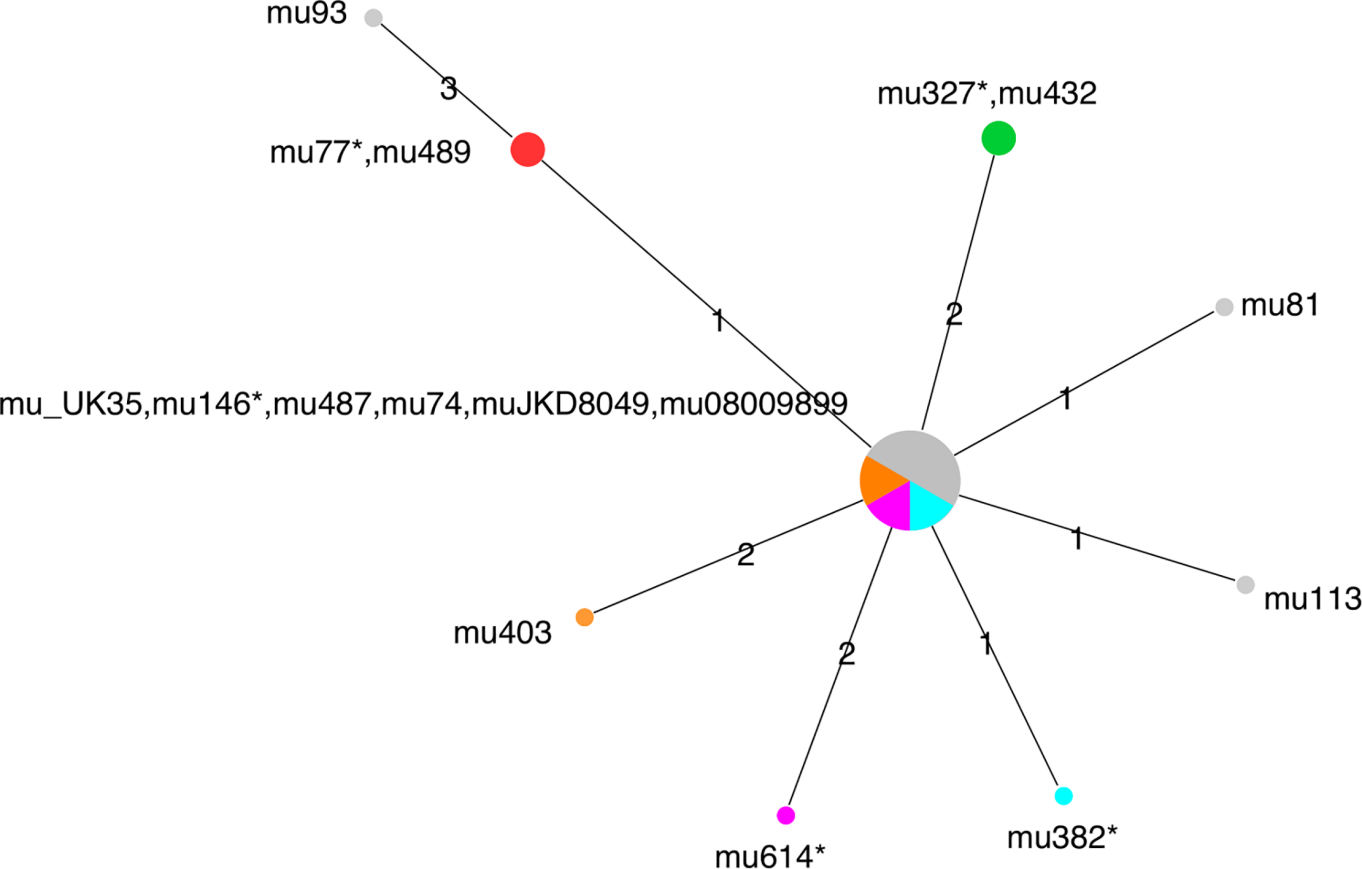
Median joining network of 10 *M*. *ulcerans* isolates derived from five recurrent cases of *M*. *ulcerans* disease in south-eastern Victoria. Node colours represent paired isolates, *grey* nodes represent six unrelated isolates also from *M*. *ulcerans*. The size of each node is proportional to the number of isolates with identical genotypes. Edges are labelled with the number of SNPs between each node. Asterisks show the primary (or initial) isolate.

Whole genome pairwise comparisons of the paired isolates revealed close genetic similarity between pairs (Fig 2). Indeed, based on our SNP analysis the paired isolates from the patients #3 (mu77/mu489) and #2 (mu327/mu432) were genetically identical (Fig 2, Table 3). In contrast, paired isolates from patients #1, #4 and #5, contained SNP differences between each pair (Fig 2, Table 3). To put this genetic variation in context, we also performed SNP analysis on an additional six unrelated human *M*. *ulcerans* isolates from the same endemic area. Three of the six isolates (mu74, muJKD8049, mu08009899) were genetically identical to each other following SNP analysis (Fig 2). Three isolates (mu146, mu_UK35 and mu487) from the paired cases were also genetically identical to these isolates demonstrating that even apparently unrelated isolates can share a common genotype. Furthermore, this genotype appeared the most dominant within the Bellarine Peninsula isolates we examined. Within two of the three pairs that contained this ‘common’ genotype (#1 and 5), the primary isolate was more genetically divergent from this ‘common’ genotype compared to the second (reoccurring) isolate. The time interval between recurrent lesions did appear to greatly influence the number of SNP differences between the isolates.

**Table 3:** Genetic changes between paired isolates. ‘Position’ references to the reference genome Agy99 (NC_008611).

Pair number	Isolates	No. SNPs	Position and nucleotide change	Gene (amino acid change)	Proposed re-infection or relapse
1	mu614 vs. mu_UK35	2	5258932 (AT) unique insert in mu614	intergenic	Re-infection
2	mu327 vs. mu432	0			Relapse
3	mu77 vs. mu489	0			Re-infection with same genotype
4	mu146 vs. mu403	2	4590438 (G/C) unique to mu403 328887 (T/G) unique to mu403	FdxB (Gly/Ala) Acyl-CoA dehydrogenases (Lys/Thr)	Re-infection
5	mu382 vs. mu487	1	5352860 (A/G) unique to mu382	FadD12_2 (Cys/Arg)	Re-infection

## Discussion

Our study has shown that Buruli ulcer has a low recurrence rate in treated Australian patients with an assumed cure living in an endemic region. This provides important prognostic information for patients and their health providers, and may help alleviate the often substantial fears that patients have of becoming reinfected once their initial lesion has been cured. Although the low risk is reassuring, the fact that it can occur means that patients and clinical staff need to be educated and aware of this possibility, so that any recurrent lesions are assessed and diagnosed early when lesions are small, enabling less complex treatment with better outcomes [5]. It is also important to recognise that recurrent lesions can occur many years later and commonly occur on completely different regions of the body compared to the initial lesion. In our study we did not detect an increased risk of recurrent lesions associated with patient characteristics which included age, gender, WHO category and type of lesion, diabetes, immune suppression and the duration of symptoms prior to diagnosis. Although we did not examine host genetics, previous studies have identified genetic factors associated with increased susceptibility to *M*. *ulcerans* that may influence the risk of recurrent disease. [14,15] We would suggest future studies be performed to assess whether host genetics can predict those at risk of recurrences, or whether this is more likely determined by the intensity of re-exposure.

The whole genome sequence analysis revealed a mix of genetic relationships between isolates. Paired isolates from some patients (#2 and #3) were genetically identical, possibly suggesting either late relapse of the initial infection or re-infection from a genetically homogenous source. In the case of patient #3, the extended time between recurrence (46 months), the fact that the patient received highly effective treatment, and the fact that the lesions were identified in different body areas (right forearm and left ankle), suggests that re-infection from a genetically homogenous source was more likely. While it’s hard to estimate the degree of genetic change that would occur during a latency period *in vivo*, we assume that some mutations would occur with longer periods (particularly 46 months). In contrast, the isolates from patient #2 –also genetically identical–were only separated by 12 months, and occurred on the same body region. In this case, a late relapse of the initial infection would appear more likely.

There were genetic differences between three of the paired isolates (patients #1, #4, and #5) which can be interpreted in two ways. Firstly, it’s possible that they are the result of re-infection from a genetically heterogeneous population. In support of this hypothesis, our previous research examining family clusters of *M*. *ulcerans* cases in Australia suggests that exposure risk to *M*. *ulcerans* is short-term and may not necessarily be from a genetically homogeneous source [10]. However, given that *M*. *ulcerans* is highly clonal in Australia, with only minor genetic variation [13,16], it is expected that some re-infection cases will also be from genetically identical sources. The case of patient #3, discussed above, would be an example here. The second possible explanation is that the bacterium genetically evolves during its latency period *in vivo* and thus the cases represent late disease relapse despite a small number of SNP differences. In the case of patients #1 and #5 this latter hypothesis cannot be ruled out, but seems unlikely as in both cases the primary (first) isolate had genetically diverged more from the ‘common’ dominant genotype compared to the second isolate. This is further supported in patient #1 by the long duration between lesions (44 months) and in patient #5 by the recurrent lesion being situated on a completely different body area and the initial treatment being highly effective for curing BU. Combined, these findings suggests that re-infection with a different genotype was the most plausible explanation for the #1 and #5 cases.

In comparison with the other known study by Eddyani et al. from Africa [6] that looked at recurrent BU cases post treatment between 1989 and 2010 using whole genome sequencing, their recurrence rate (100/4951 cases; 2.0%) was similar to ours (1.6%). However this study included recurrent lesions occurring from 6 months following treatment meaning their recurrence rate according to our definition (≥ 12 months) would have been lower. With information from clinical, treatment and epidemiological data supported by whole genome sequencing, 80% of our cases were classified as re-infection whereas 75% of their cases were classified as relapse. In the African study, none of the three cases classified as relapse received effective antibiotics against *M*. *ulcerans*, putting them at higher risk of relapse [7], and in 2 of the three cases the isolates were genetically identical. The third relapse isolate differed by only 1 SNP and occurred on the same body region within a short time interval (9.5 months). In their single case classified as re-infection, the second lesion was on a separate body area and the isolate had a 20 SNP difference compared to the original one. Thus their interpretations were similar to ours whereby the one case we classified as relapse (#2) had a genetically identical isolate on the same region of the body within a short time interval (12 months), whereas those classified as re-infection had a combination of either being genetically distinct isolates (#1,4,5), on separate body areas (#3,4,5), having had highly effective treatment (#3 and 5) or having a long time interval between cases (#1,3,4). From both studies it is evident that whole genome sequencing can be a useful tool in helping to clarify the likelihood of BU relapse versus re-infection post treatment, as has been the case with tuberculosis [17].

The two recurrent cases who did not have paired isolates available for WGS (#6 and 7) were classified as re-infections based on a combination of separate body areas (#6 and 7), highly effective treatment (#7) and having a long time interval between cases (#6 and 7). Additionally, our data suggesting that all recurrent cases which occurred more than 12 months after treatment commenced were classified as re-infections, and the only one occurring after 12 months was classified as disease relapse, would support our previous clinical definitions that treatment failure occurs when a recurrent lesion appears within 12 months of commencing treatment[5].

If, as suggested by our study, most recurrent cases result from re-infection, then at least for a proportion of treated patients acquired protective immunity against the development of recurrent *M*. *ulcerans* disease does not develop following an initial infection. Interestingly, the rate of recurrence (2.81 cases/year/1000 population) was similar to the estimated risk of infection in the general population of the Bellarine Peninsula (0.85–4.04 cases/year/1,000 population)[18], suggesting that there may be no significant risk reduction against future infection for previously treated patients. This is in contrast to a study from Uganda in the 1970s which suggested an 88% protective effect over 4 years against recurrent *M*. *ulcerans* disease in those with a prior history of the disease.[19]

A limitation of our study is that we relied on self-presentation or referral to our health service for diagnosis of recurrent lesions more than 12 months after treatment commenced and therefore there is a risk that some recurrent lesions were not captured in our study. However, as we are the only specialised health service in our region managing *M*. *ulcerans* it is likely that any recurrent lesions in patients would have be managed at Barwon Health and therefore we feel the risk of missing recurrent lesions would be small. Additionally, as the incidence of *M*. *ulcerans* in the Bellarine Peninsula has fallen in recent years,[20] if this reduction relates to reduced environmental pressure for infection we may have underestimated the risk of recurrent lesions that would occur if the pressure had remained constant. It is also recognised that the number of recurrent cases where isolates had WGS performed was small meaning our results need to be interpreted with some caution. Further research involving WGS of more isolates from recurrent cases should be performed to further validate these findings.

## Conclusions

There is a low incidence of recurrent Buruli ulcer in treated Australian patients living in endemic regions and the risk is similar to the estimated risk of primary infection within the general population of the endemic area. The majority of recurrent lesions appear to result from re-infection suggesting that for a proportion of treated patients lifelong immunity against *M*. *ulcerans* re-infection does not develop.
